# Acceptability and appropriateness of a clinical pathway for managing anxiety and depression in cancer patients: a mixed methods study of staff perspectives

**DOI:** 10.1186/s12913-021-07252-z

**Published:** 2021-11-17

**Authors:** Phyllis Butow, Heather L. Shepherd, Jessica Cuddy, Marnie Harris, Sharon He, Lindy Masya, Mona Faris, Nicole M. Rankin, Philip Beale, Afaf Girgis, Brian Kelly, Peter Grimison, The ADAPT Program Group, Philip Beale, Phyllis Butow, Josephine Clayton, Jessica Cuddy, Fiona Davies, Haryana Dhillon, Mona Faris, Liesbeth Geerligs, Afaf Girgis, Peter Grimison, Tom Hack, Marnie Harris, Sharon He, Brian Kelly, Patrick Kelly, Laura Kirsten, Toni Lindsay, Melanie Lovell, Tim Luckett, Lindy Masya, Michael Murphy, Jill Newby, Don Piro, Nicole Rankin, Joanne Shaw, Tim Shaw, Heather Shepherd, Rosalie Viney, Jackie Yim, Joanne Shaw

**Affiliations:** 1 Psycho-Oncology Co-operative Research Group (PoCoG), School of Psychology, The University of Sydney, Sydney, NSW Australia; 2 Faculty of Medicine and Health, The University of Sydney, Sydney, NSW Australia; 3 Cancer Services for the Sydney Local Health District, Incorporating Royal Prince Alfred, Concord and Canterbury Hospitals, Campsie, NSW Australia; 4 Ingham Institute for Applied Medical Research, South Western Sydney Clinical School, University of New South Wales, Kensington, NSW Australia; 5 School of Medicine & Public Health, University of Newcastle, Callaghan, NSW Australia; 6 Chris O’Brien Lifehouse, Camperdown, NSW Australia

**Keywords:** Anxiety and depression, Cancer, Clinical pathway, Implementation, Routine care

## Abstract

**Background:**

Clinical pathways (CPs) can improve health outcomes, but to be sustainable, must be deemed acceptable and appropriate by staff. A CP for screening and management of anxiety and depression in cancer patients (the ADAPT CP) was implemented in 12 Australian oncology services for 12 months, within a cluster randomised controlled trial of core versus enhanced implementation strategies. This paper compares staff-perceived acceptability and appropriateness of the ADAPT CP across study arms.

**Methods:**

Multi-disciplinary lead teams at each service tailored, planned, championed and implemented the CP. Staff at participating services, purposively selected for diversity, completed a survey and participated in an interview prior to implementation (T0), and at midpoint (6 months: T1) and end (12 months: T2) of implementation. Interviews were recorded, transcribed and thematically analysed.

**Results:**

Seven metropolitan and 5 regional services participated. Questionnaires were completed by 106, 58 and 57 staff at T0, T1 and T2 respectively. Eighty-eight staff consented to be interviewed at T0, with 89 and 76 at T1 and T2 (response rates 70%, 66% and 57%, respectively). Acceptability/appropriateness, on the quantitative measure, was high at T0 (mean of 31/35) and remained at that level throughout the study, with no differences between staff from core versus enhanced services. Perceived burden was relatively low (mean of 11/20) with no change over time. Lowest scores and greatest variability pertained to perceived impact on workload, time and cost. Four major themes were identified: 1) Mental health is an important issue which ADAPT addresses; 2) ADAPT helps staff deliver best care, and reduces staff stress; 3) ADAPT is fit for purpose, for both cancer care services and patients; 4) ADAPT: a catalyst for change. Opposing viewpoints are outlined.

**Conclusions:**

This study demonstrated high staff-perceived acceptability and appropriateness of the ADAPT CP with regards to its focus, evidence-base, utility to staff and patients, and ability to create change. However, concerns remained regarding burden on staff and time commitment. Strategies from a policy and managerial level will likely be required to overcome the latter issues.

**Trial registration:**

The study was registered prospectively with the ANZCTR on 22/3/2017. Trial ID ACTRN12617000411347. https://www.anzctr.org.au/.

**Supplementary Information:**

The online version contains supplementary material available at 10.1186/s12913-021-07252-z.

## Background

Clinical pathways (CPs) are standardised, evidence-based multidisciplinary management plans, which identify an appropriate sequence of clinical interventions, timeframes, milestones and expected outcomes for one or more patient groups [1]. CPs are more detailed than clinical guidelines, as they are operational in nature. CPs are increasingly being used in healthcare to improve outcomes, such as better patient quality of life and care [2, 3], increased hospital efficiency [4, 5], decreased operations costs [6, 7], reduced length of stay [8] and decreased mortality rates [9]. However, despite these reported benefits, CPs will not impact patient outcomes unless they are sustainable. Two key factors impacting CP sustainability are their acceptability and appropriateness [10].

Our group developed a CP for screening, assessment and management of anxiety and depression in adult cancer patients (the ADAPT CP), based on an evidence review and wide stakeholder input [11, 12]. Utilising a stepped care model, the ADAPT CP involves screening via patient reported outcomes at regular intervals, triage to one of five steps based on symptom severity, and management tailored to severity step (from universal care and self-management for mild anxiety/depression to specialist psycho-oncology care for severe anxiety/depression). Mid-management review is recommended with change of approach if required. The ADAPT CP provides evidence-based recommendations on appropriate staffing, and the content and timing of interventions for each step [11]. Resources were developed to support the CP, including an online portal [13] to operationalise and standardise as many processes as possible, to increase efficiency and minimise staff time and burden, as well as staff and patient education materials, accessible via the portal.

We implemented the ADAPT CP in a cluster randomised controlled trial (CRCT) in 12 oncology services in NSW Australia [14]. The ADAPT CRCT is evaluating the impact of two levels of implementation strategy intensity on staff adherence to the CP (described below). Based on Proctor and colleagues’ influential 2011 article [15] which defined a conceptual framework for implementation outcomes, we explicitly outlined measures of success for the ADAPT CRCT prior to study commencement [16]. Two key outcomes defined were acceptability and appropriateness. Focusing on the CP, acceptability was defined as “cancer staff perceptions of the ADAPT CP intervention and its components as agreeable, palatable, or satisfactory.” Appropriateness was defined as “the extent to which cancer staff believe that the ADAPT CP and its resources has fit, relevance and compatibility at the level of their setting, their role and the needs of their patients.”

Proctor et al. [15] recommended that acceptability and appropriateness be assessed based on diverse stakeholders’ direct experience with various dimensions of the intervention, such as its content, complexity, or comfort. Furthermore, they note these outcomes are dynamic, changing with experience, and should thus be assessed longitudinally throughout the implementation phase. Thus, the aim of this paper was to describe staff perceptions of the acceptability and appropriateness of the ADAPT CP, assessed longitudinally over the 12-month implementation period. Second, we aimed to explore differences between trial arms (core versus enhanced) in these outcomes to determine whether implementation intensity impacts perceptions of the CP itself.

## Methods

### Study design and setting

For the ADAPT CRCT, eligible services were public or private health services providing cancer care for at least 100 patients per year [14]. In Australia, cancer services provide both inpatient and outpatient care. Chemotherapy and radiation therapy is typically provided through tumour specific outpatient services. Potential CRCT sites were purposively selected to provide diversity in urban versus regional settings, and size of patient load. Thirteen services declined participation as they were currently undergoing physical or organisational change and did not feel able to commit to further change. Once the required number of services (12 - based on the power calculation for the main trial) [14] was achieved, site recruitment ceased.

### Study procedures

The ADAPT CP and trial processes have been described in detail elsewhere [14], but in brief, at each site, after one or more champions and a multidisciplinary lead team were appointed, 6–8 engagement meetings were held to tailor the pathway to local resources and preferences and establish service workflows to implement the CP. All services had access to the ADAPT resources described above, and received education and training in the CP and portal and marketing support (newsletters, posters, roadshows, emails from service champions) before implementation. The CP was then implemented for 12 months, with the CP offered to patients as part of routine care. See Additional file 1 for Consort flow diagram.

### Study arms

Following the CP launch, the two arms diverged. Services in the core implementation arm were able to initiate contact with the research team if help was required, were provided monthly portal reports (detailing CP activities at the patient, clinician and service level), but were otherwise left to their own devices. Services in the enhanced implementation arm received proactive contact from the research team throughout the implementation period, with monthly review meetings at which data on service CP use was presented and discussed, and ongoing strategies such as newsletters and posters were provided to staff to encourage engagement.

### ADAPT CP process

During the implementation (as outlined in site workflows), selected staff were responsible for registering patients on the ADAPT portal; patients then received an automated email to screen or were flagged within the ADAPT portal as due for screening using a validated tool (Distress Thermometer (DT) [17] or Edmonton Symptom Assessment Scale Revised (ESAS-r) [18] as selected by the service). Scores were automatically calculated; if patients scored above cut-off (DT ≥4 or ESAS-A ≥ 3 and ESAS-D ≥ 2, respectively) they were directed to complete the Hospital Anxiety and Depression Scale (HADs) [19]. Patients were then allocated a step (1–5) reflecting the severity of their anxiety/depression, using published and consensus-derived cut-offs on the HADs [19] (0–3, 4–7, 8–10, 11–14, 15+ for minimal, mild, moderate, severe and very severe, respectively). If step 2 or above, an email was sent by the portal to responsible staff to prompt a meeting with the patient for further assessment, and provide recommendations for management based on the patient’s step (see Additional file 2 for management recommendations for each step). If appropriate, staff accessed a referral template on the portal to refer the patient to other specialist mental health clinicians for further management. Patients and clinicians received a mid-therapy check of progress, and post-therapy, patients were cued to screen again at approximately 3-monthly intervals (interval-period selected by each service). Staff were responsible for recording their actions in the portal and responding to email prompts in a timely fashion.

### Staff participants

The goal was to obtain a cross-sectional snap shot of staff perceptions at each time-point. The sample comprised lead team attendees at each of the monthly meetings, as well as staff who interacted in any capacity with ADAPT. An email was sent to all staff who had registered on the portal, attended training, or had an ADAPT role, as nominated by a Champion, to invite them to participate by completing questionnaires and interviews. Non-members of the lead team were included to ensure representation of views of staff on the ground actually enacting the ADAPT CP. Staff were assured of confidentiality. Non-responders were followed up with reminder emails twice. However, in response to staff movement and changes, staff who had employment contracts of less than 6 months were excluded.

### Data collection

#### Questionnaires

Staff were invited to complete a questionnaire just prior to the ADAPT CP implementation, but after the engagement meetings (T0), and again at 6 months (T1) and 12 months (T2) into the 12-month implementation. Demographic and professional details were elicited. Staff then completed 13 study-developed items to assess acceptability/appropriateness (Additional file 3). Participants responded to items on a 5 point Likert scale (agree completely to disagree completely). Factor analysis revealed two stable factors; scores were summed, with higher scores indicating greater acceptability and appropriateness.

#### Interviews

At each assessment point (T0, T1 and T2), a subset of staff, purposively selected, at each service were invited to participate in a semi-structured telephone interview exploring amongst other issues, their response to the tailoring of the ADAPT CP to their service context. The interviews were conducted by three female qualitatively trained researchers with no direct involvement with ADAPT CP delivery. Interviews were audio-recorded and transcribed.

### Analysis

Quantitative data were analysed in Stata version 17. Summary statistics were generated, and the two arms of the study compared using Mann-Whitney test due to small and uneven sample sizes, and because data are not independent. Interviews were coded in NVivo12 [20]. T0, T1 and T2 interview data were thematically analysed [21] to identify themes regarding acceptability of the ADAPT CP. Two researchers coded an initial six transcripts to develop a draft coding tree, which was then discussed with a third researcher and refined. They then independently coded interviews line-by-line, with any differences resolved through consensus. Similarities and differences in codes were examined to develop initial themes, which were then reviewed to develop higher order themes.

Framework analysis [22] was applied, with themes and quotes mapped against implementation arm and assessment to explore systematic differences according to randomisation and timing. Results were mapped to the the Consolidated Framework for Implementation Research (CFIR) [23], a consolidated framework identifying factors influencing implementation success.

## Results

*Services:* Twelve cancer services (7 located in metropolitan and 5 in regional areas) participated in the ADAPT CRCT. Ten services were publicly funded, one was a public/private partnership, and one was privately funded. Five services had already conducted screening for anxiety and depression within the past 12 months using a validated tool (see Table 1 for study site characteristics).

**Table 1:** Site characteristics

Site ID	Allocated Study Arm	Site Location	Funding Type	Number of patients seen per 3-month period	Number of departments included	Treatment modality departments included	Tumour Streams included	Number of streams included	FTE Psycho-social staff	Screening History in past 12 months
1	Core	Major city	Public	≥100	3	Med Oncology Rad Oncology Haematology	All	≥3	0.8	Yes
2	Enhanced	Inner regional	Public	< 100	4	Med Oncology Rad Oncology Haematology Surgical	All	≥3	0.6	No
3	Core	Inner regional	Public	< 100	1	Med Oncology	All	≥3	0.6	No
4	Enhanced	Major city	Public	≥100	2	Med Oncology Surgical	Gastro-intestinal	1	2.4	No
5	Enhanced	Inner regional	Public	< 100	3	Med Oncology Rad Oncology Haematology	All	≥3	1	Yes
6	Enhanced	Major city	Public	≥100	2	Med oncology Haematology	All	≥3	7.9	No
7	Enhanced	Major city	Public	≥100	1	Surgical	Upper GI	1	2.4	Yes
8	Enhanced	Major city	Public	< 100	3	Med Oncology Rad Oncology Haematology	All	≥3	5	Yes
9	Enhanced	Major city	Public	≥100	1	Haematology	Lymphoma, acute leukemia, multiple myeloma	≥3	2.4	No
10	Enhanced	Major city	Public	≥100	3	Med Oncology Rad Oncology Surgical	Head & Neck	1	4	No
11	Core	Major city	Public and Private	≥100	1	Med Oncology	Sarcoma, Gynae	2	6.9	Yes
12	Core	Major city	Private	≥100	1	Med Oncology	All	≥3	0.9	No

### Quantitative data

At T0, 106 staff completed questionnaires (30 in core, 76 in enhanced services). At T1, 58 staff (23 in core and 35 in enhanced services) completed questionnaires, and at T2, 57 (26 in core, 31 in enhanced services) did so. Reduced numbers over time were likely due primarily to staff movement and lack of time. See Table 2 for participant characteristics.

**Table 2:** Staff survey participants: Demographic and professional characteristics

	T0 (***n*** = 106)		T1 (***n*** = 58)		T2 (***n*** = 57)	
	n	%	n	%	n	%
Age Range (in years)						
18–25	3	2.8	1	1.7	1	1.8
26–50	73	68.9	40	69.0	44	77.2
51–75	30	28.3	17	29.3	12	21.1
Gender						
Female	91	85.8	50	86.2	51	89.5
Male	15	14.2	8	13.8	6	10.5
^a^Role						
Nursing staff	47	44.3	21	36.2	22	38.6
Medical staff	17	16.0	10	17.2	6	10.5
Allied health & clinical trials staff	9	8.5	1	1.7	2	3.5
Administrative, technical support and non-clinical managers	11	10.4	7	12.1	5	8.8
Psycho-social staff	21	19.8	18	31.0	15	26.3
Unspecified role	1	0.9	1	1.7	1	1.8
Missing	0	0.0	0	0.0	6	10.5
Employment Status						
Full-Time	70	66.0	36	62.1	39	68.4
Part-Time	36	34.0	22	37.9	18	31.6
Years of employment in current role						
< 1 year	12	11.3	2	3.4	2	3.5
1–5 years	52	49.1	33	56.9	34	59.6
6–20 years	35	33.0	21	36.2	20	35.1
21>	7	6.6	2	3.4	1	1.8
Language spoken at home						
English	85	80.2	48	82.8	45	78.9
^b^Other	21	19.4	8	13.7	12	21.3
Missing	0	0.0	2	3.4	0	0.0
Country of birth						
Australia	72	67.9	36	62.1	41	71.9
^c^Other	34	30.7	22	37.7	15	26.3
Missing	0	0.0	0	0.0	1	1.8
Aboriginal or Torres Strait Islander						
No	106	100.0	57	98.3	57	100.0
Yes, Aboriginal	0	0.0	1	1.7	0	0.0

^a^Roles included in the categories:

Nursing Staff: Nurse- RN/AIN, CNS, CNE Care Coordinator, CNC, NUM, Nurse Practitioner

Medical Staff: Oncologist, Haematologist, Psychiatrist, Registrar, Medical oncology Fellow

Allied Health & Clinical Trials Staff: Speech pathologist, Clinical Trials

Admin, technical support & non-clinical managers: Admin, IT staff, Volunteer, Clinical Support Officer, Management, Program Coordinator, Practice Manager

Psychosocial staff: Psychologist, Psychologist Intern, Social Worker, Counsellor

^b^Other languages spoken at home: Cantonese, Mandarin, Indonesian, Malayalam, Spanish, Tagalog, Arabic, Filipino, French, Portuguese, Greek, Italian, Korean, Macdeonian, Tamil, Sindhi, Vietnamese, Croatian, Bosnia, Serbian, Iban, Malaysian

^c^Other countries of birth: Bosnia and Herzegovina, China, Hong Kong, India, Indonesia, Italy, Korea, Macdeonia, Malaysia, New Zealand, Pakistan, Peru, Philippines, Portugal, South Africa, Sri Lanka, United States of America, UK, Vietnam

An exploratory factor analysis was conducted on the acceptability/approriateness items to assess their latent structure. Two factors (positive acceptability/appropriateness (or benefit) and negative acceptability/appropriateness (or burden)) were extracted (eigenvalues > 1); these accounted for 84.5% of the variance and were weakly correlated (.29). Using Varimax rotation (with Kaiser normalisation), most items factored well (Kaiser-Meyer-Olkin (KMO) = all items >.60, except for item 7 (0.53)). One item did not load on to any factor and was excluded. Internal consistency was acceptable (Cronbach’s Alpha for Factor 1 = 0.86 and for Factor 2 = 0.71).

**Fig. 1:**
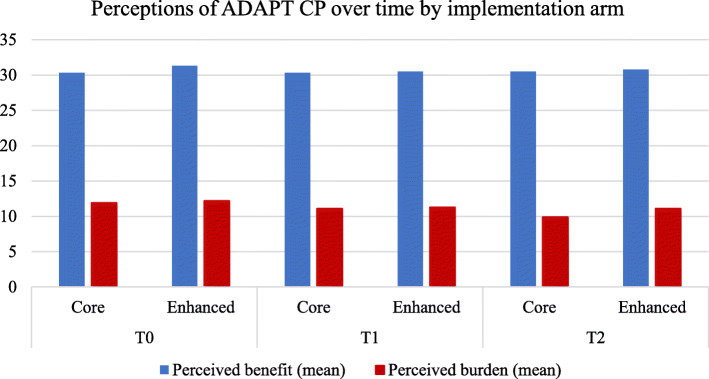
Perceived benefit and burden of ADAPT CP by implementation arm

Total benefit and burden scores were calculated by summing scores within each factor (Fig. 1). Mean perceived *burden* scores were moderate (12.6/20) dropping slightly across time (11.2 (T1) and 10.5 (T2)), with no differences between trial arms at any timepoint. Highest scores (with greatest variability) pertained to items addressing perceived impact on workload, time and cost. Mean perceived *benefit* scores were high at T0 (30.8/35) remaining very consistent over time, with no difference between arms.

### Qualitative data

Eighty-eight, 89 and 76 staff participated in interviews at T0, T1 and T2 respectively. The overall response rate across all time points was 64%, however response rates decreased slightly over time (70% at T0, 66% at T1, and 57% at T2). A total of 122 unique staff participated; 44 staff (36%) were interviewed at all three time points, 43 (35%) at two time points, and 35 (29%) at one. Average interview duration was 22 min at T0 and 24 min at T1 and T2. Participants had an average of 6 years of experience in their role (range 0–33). For full demographic details of interview participants, see Table 3.

**Table 3:** Staff interview participants: Demographic and professional characteristics

	T0 (***n*** = 88)		T1 (***n*** = 89)		T2 (***n*** = 76)	
	n	%	n	%	n	%
Age Range (in years)						
18–25	2	2.3	2	2.2	3	3.9
26–50	61	69.3	67	75.3	48	63.2
51–75	23	26.1	16	18.0	22	28.9
Missing	2	2.3	4	4.5	3	3.9
Gender						
Female	75	85.2	73	82.0	66	86.8
Male	13	14.8	16	18.0	10	13.2
^a^Role						
Nursing Staff	33	37.5	34	38.2	26	34.2
Medical Staff	12	13.6	13	14.6	8	10.5
Allied Health and Clinical Trials Staff	6	6.8	4	4.5	8	10.5
Administrative, technical support and non-clinical managers	15	17.0	12	13.5	13	17.1
Psycho-social Staff	22	25.0	26	29.2	21	27.6
Employment Status						
Full-time	57	64.8	58	65.2	49	64.5
Part-time	27	30.7	26	29.2	24	31.6
Part-time, independent contractor	2	2.3	0	0.0	0	0.0
Full-time, independent contractor	0	0.0	1	1.1	0	0.0
Missing	2	2.3	4	4.5	3	3.9
Language spoken at home						
English	77	87.5	74	83.1	65	85.5
^b^Other	9	10.2	11	12.4	8	10.6
Missing	2	2.3	4	4.5	3	3.9
Country of birth						
Australia	62	70.5	58	65.2	52	68.4
^c^Other	24	27.2	27	30.3	21	27.7
Missing	2	2.3	4	4.5	3	3.9
Aboriginal or Torres Strait Islander						
No	85	96.6	84	94.4	73	96.1
Yes, Aboriginal	1	1.1	1	1.1	0	0.0
Missing	2	2.3	4	4.5	3	3.9

^a^Roles included in the categories:

Nursing Staff: Nurse- RN/AIN, CNS, CNE Care Coordinator, CNC, NUM, Nurse Practitioner

Medical Staff: Oncologist, Haematologist, Psychiatrist, Registrar, Medical oncology Fellow

Allied Health & Clinical Trials Staff: Speech pathologist, Clinical Trials

Administrative, technical support & non-clinical managers: Administrative, IT staff, Volunteer, Clinical Support Officer, Management, Program Coordinator, Practice Manager

Psychosocial staff: Psychologist, Psychologist Intern, Social Worker, Counsellor

^b^Other languages spoken at home: Mandarin, Cantonese, Indonesian, Portuguese, Spanish, Tagalog, Malayalam

^c^Other countries of birth: UK, Canada, China, India, Indonesia, Brazil, Kenya, Hong Kong, Philippines, Sri Lanka, South Africa, Peru, New Zealand

Qualitative analysis of staff interviews revealed four over-arching themes: 1) Mental health is an important issue which ADAPT addresses; 2) ADAPT helps staff deliver best care, and reduces staff stress; 3) ADAPT is fit for purpose, for both the cancer care setting and patients; 4) ADAPT: a catalyst for change. Themes generally remained stable over time and did not differ between study arms. Change and differences are noted where they occurred. Quotes are identified by arm of study: enhanced (E) versus core (C); profession, oncology service (S1–12); personal ID (PID); and time of assessment (T0–2). Additional quotes are provided in Additional file 4.

#### 1. Mental health is an important issue which ADAPT addresses

Almost all participants supported the importance of identifying and managing patients’ anxiety and depression. They believed these to be common co-morbidities which, if not addressed, make it significantly harder for patients to cope with their disease, its treatment and survivorship.

*“everybody that has a cancer diagnosis will have some degree of anxiety and will need some support, and when they need a lot of support that needs to be identified quickly...”* (C_ADMIN_S01P07T1)

##### 1.1 It is our responsibility to address anxiety and depression

Participants believed that addressing anxiety and depression was part of holistic, patient-centred care. Some staff felt that at their site, psychological morbidity was not addressed as well as it could be, and therefore ADAPT would provide a much-needed opportunity to remedy this gap.

*“It’s been one of the areas that’s been quite lacking, as far as, support for patients in this area. So I think that’s something that everybody is very excited about.”* (E_NURS_S02P09T0)

##### 1.2 ADAPT is evidence-based

Staff believed that ADAPT was firmly based on good evidence, and trusted the research team’s expertise; this was primarily emphasised at T0.

*“I think it’s really useful and very evidence-based … it’s actually reassuring that what we’re doing is actually evidence-based.”* (E_NURS_S08P01T0)

##### 1.3 ADAPT is systematic and will ensure patients are not missed

Staff noted that ADAPT would ensure all patients who are distressed and need support are identified, with none “*falling between the cracks*”. Staff noted that some patients have no visible signs of distress and therefore are hard to identify. While current practice picked up some cases, it was felt to be “*adhoc”*, “*informal”* and “*uncoordinated”*; some patients were missed because key staff were absent. Furthermore, the repeat screening step of the ADAPT CP was perceived to ensure adequate follow-up, to identify late-emerging symptoms.

*“At the moment, we deal with anxiety and depression but it’s really ad hoc - sometimes we’re hit and miss. Because there could be patients that don’t show they're anxious or have any depression … and they just soldier on. This way … we’re going to pick up a lot more.”* (E_ADMIN_S02P05T0)

*“We've picked up some things that wouldn't have, ordinarily, been picked up … and been able to provide services for those patients.”* (E_AH_S03P06T2)

However, some staff believed ADAPT to be unnecessary at their site, as their psychosocial care and referral processes already ensured that all patients who required support were identified.

*“I think in my service in particular it is not exactly necessary … we are finding that a lot of the patients that are screening positive through the portal are already in touch with our psychology service.”* (C_NURS_S11P03T1)

While in some sites this perception changed over time, in others staff remained confident that ADAPT was not needed throughout the implementation period.

*“So far all of the patients who’ve been identified are patients who are already known to us. So, so far the utility of it probably hasn’t been particularly helpful.”* (C_PSYCH_S11P01T1)

##### 1.4 ADAPT will “nip problems in the bud”

Some staff noted that ADAPT ensured early identification of distress in patients, thus preventing these symptoms from progressing to a full-blown clinical problem. This was seen as a way of reducing suffering, as well as reducing need for staff and service resources.

*“So if we have a system to certify the patient needs that help and refer to them early rather than late. And then, I think, make them, the journey a little bit easy.”* (C_NURS_S12P05T2)

However, some staff (primarily in surgical wards) felt ADAPT was less nimble than their current processes, potentially making it more likely that patients would not be identified until later in their treatment trajectory. This was primilarly because of the additional administrative steps involved in working with ADAPT’s online portal.

*“so it’s just that ADAPT– it’s just a slow moving beast. Whereas … the protocol that we had for distress … it allowed for quick reactions and referrals … rather than screen in a month and we’ll see where you’re up to … that just doesn’t seem to be a good fit for the surgical context.”* (E_PSYCH_S07P01T1)

##### 1.5 ADAPT will empower patients to self-manage

Staff felt ADAPT, by normalising mental health needs, reinforcing the importance and validity of seeking help, and increasing awareness of mental health supports available, would empower patients to firstly identify when they were anxious or depressed, and secondly to seek appropriate help. On the whole, ADAPT was perceived as being highly acceptable.

*“People can often put on a brave face … until … the actual conversation is facilitated … I feel the pathway’s actually brought a lot of opportunities for people to get help that may … not have reached out for help otherwise. Or they didn’t know how to reach out for help.”* (C_NURS_S12P01T1)

However, some staff felt that ADAPT could not overcome the stigma of mental health.

*“To be honest … I think the last report said that we only had one person screened and I, personally, just think it’s our area, our demographics. It’s the stigma that goes with it. And I don’t think that will ever change.”* (E_NURS_S04P02T1)

#### 2. ADAPT helps staff deliver best care, and reduces staff stress

Many staff thought that ADAPT was also acceptable and appropriate for staff. They felt ADAPT raised their awareness of psychosocial issues, and provided them with new skills and a well-planned, evidence-based intervention, ultimately improving their capacity to provide the best patient care. Having tried a different approach with ADAPT, staff felt they would have the confidence to continue raising mental health in routine care.

*“Two weeks ago the patient re-screened … and she screened high still … I was able to communicate with the doctor and offer her suggestions of what to do, and her treatment did change and she did have a better fortnight because of that.”* (C_NURS_S12P01T1)

##### 2.1 Less stressed patients mean less stressed staff

Staff felt their own work would be made easier and less stressful, as they would be informed upfront of patient concerns so they were not on “*their backfeet*” in addressing them, and patients would be less likely to present in crisis.

*“They aren't asked early enough and then before we know it the patients have all these social issues at home and we're kind of on the back foot, we're not proactive about addressing it, we're just kind of reacting, so I definitely think the questions need to be asked.”* (C_NURS_S01P06T1)

##### 2.2 A multidisciplinary approach is enriching and supportive

Finally, staff felt that by raising the profile of mental health, creating more rigorous triage and establishing a whole-of-team approach, some staff would be better supported to deliver this care, while others felt that working with multidisciplinary colleagues enriched their working life.

*“I think the advantage will be that it will cut my load down … they can do the work themselves … through the resources, so I think that’s excellent.”* (E_PSYCH_S05P01T0)

*“ … the referral pathways are working... [prior to ADAPT] I would get contacted and then I would be the one trying to figure out what to do next and who else to involve.”* (C_MED_S01P09T1)

#### 3. ADAPT is fit for purpose, for both the cancer care setting and patients

Staff commented both positively and negatively regarding the fitness for purpose of ADAPT within the cancer care setting, and for their patients.

##### 3.1 Appropriateness of ADAPT for staff

Some staff noted over time that ADAPT fitted well, or had been made to fit well, into the existing workflow, complementing existing processes without adding significant burden. Some staff were surprised at how easy the process had been, while others found this took more time and effort. Having only a small number of patients score in the severe range of anxiety and depression made ADAPT more manageable.

*“the way that I see it is, um, that it's not going to increase workloads, it's just going to formalise workloads. So what we're already doing will just be documented better.”* (C_AH_S03P06T0)

Sites where a pathway for psychosocial care and/or multidisciplinary collaboration were already in place, where minimal changes were required to accommodate ADAPT, were more likely to report positive experiences with workflow. In other sites, siloed clinicians or services made this a harder adjustment. Similarly, where staff felt they already had appropriate skills ADAPT was easier to accommodate, versus where additional training was required.

*“We don’t exactly know how to fit it into our normal routine just yet … it’s something new that we’ll have to adjust into our workflow*. (E_NURS_S08P01T0)

In some sites, despite very positive attitudes to the philosophy, evidence and content of ADAPT, it was perceived as too burdensome, and unsustainable. This was either due to limited capacity in existing staff, or a lack of psychosocial staff to facilitate it. Some staff felt that processes that were in place before ADAPT were a better fit for purpose and thus more appropriate.

*“But at the end of the day there's only a certain number of hours in the day, and so many things to get done … fitting everything, has been difficult.”* (C_MED_S01P09T2)

In other sites with particular characteristics (such as surgical wards where patients interacted with staff for only brief periods), ADAPT was seen as unfit for purpose, as it required ongoing follow-up and contact with patients.

*“We’ve found that it hasn’t been a very good fit for the patients that come through … They … come in, they have their operation, they go home. And so there's not that kind of ongoing continuity of care that ADAPT would be quite well suited for.”* (E_PSYCH_S07P01T1)

##### 3.2 Appropriateness of ADAPT for patients

Some staff reported that ADAPT had a very good fit for patients, who expressed gratitude and relief that they had participated in ADAPT and had their needs met.

*“All three that spoke about it with me were grateful, one of them thought she didn’t need it but took our number and when she actually, when things got a bit hairy she gave us a call which I think is excellent … ”* (E_PSYCH_S06P14T1)

Other staff noted significant resistance to screening in some patients (e.g. men and stoic patients from rural areas) which ADAPT failed to overcome. At one site, staff noted patients commented that ADAPT created a sense that they were not coping. It was not clear whether this could be overcome by improved communication when introducing ADAPT, or whether a different approach entirely was needed.

*“The men tend to be, you know, more macho and not able to really express their feelings … Country folk I think are very different your more metropolitan, city people.”* (E_NURS_S02P04T0)

*“We've had several patients who were quite distressed by the process … actually they’ve recorded that they found that quite anxiety inducing.”* (C_PSYCH_S11P01T2)

The inability of key groups of patients (e.g., those without good English skills or literacy, older patients without internet skills, and those who could not afford a computer) to access ADAPT was also noted, reducing its fitness for purpose.

*“If you don’t speak English then … we can’t help you.”* (E_NURS_S06P06T0)

*“We actually have patients that sometimes we can’t even get in contact with because they have no phone and they have no computer. They’re living in a tent or a car.”* (E_NURS_S04P02T0)

However, others predicted good uptake even in such groups, or noted that the service would try to address barriers to ensure ADAPT was fit for their patients, usually through family, staff or volunteer contact to facilitate questionnaire completion.

*“Honestly, the patients have been more willing than some staff … even the elderly patients, they might need a bit of help with these new fandangle things, but … . after the first couple of questions, they get the gist of it and they’re fine, they just fly through”* (C_AH_S03P02T2)

#### 4. ADAPT: a catalyst for change

Some staff (particularly those at the managerial level) believed that ADAPT was an opportunity to gather data, highlight gaps in the service and advocate for change and improved resources to address psychosocial care.

##### 4.1 Data to guide system improvement and provide evidence of resource needs

Participants believed that the data generated by ADAPT would provide staff and services with more information about patients’ anxiety and depression, which could be used to inform and improve patient care (“*innovative ways of doing things*” C_MED_S01P09T2) and advocate for more psychosocial staff to meet patient need. This acceptability of ADAPT at the service level is an encouraging finding and may be a contributor to longer term sustainability beyond the 12-months of implementation.

*“showing that we definitely do need … some form of psychological service here for patients. I know there’s a need, but it’s good to see it in black and white always.”* (C_NURS_S03P07T1)

*“We probably have a better idea of how many people are actually needing a psychologist assessment so that maybe that will help us make a case to have a psychologist available for our service.”* (C_MED_S01P09T1)

##### 4.2 Kickstarting improved interdisciplinary processes

At the service level, ADAPT was seen as a way to improve interdisciplinary communication, referral processes and co-operation.

*“Even within the corporate services, there seems to be, uh, not great communication. So we looked at this ADAPT trial as being quite a useful piece to help resolve that.”* (C_ADMIN_S12P08T0)

##### 4.3 Improving referral processes

Some staff commented that ADAPT prompted the service to explore new referral pathways.

*“We knew that there would always be issues with the fact that we don't have a psycho-oncologist on site. But participating in ADAPT … made us explore alternatives to having a psycho-oncologist on site.”* (C_MED_S01P09T2)

*“We did establish a pathway, and I think once the staff here saw that we could actually do something about it, we’ve even had patients referred inbetween a screening … now that we’ve got the pathway there and we have the support services mapped out.”* (C_AH_S03P02T2)

## Discussion

In this paper, we set out to describe staff perceptions of the acceptability and appropriateness of a clinical pathway (CP) for anxiety and depression prior to (T0), during (T1) and after (T2) implementation, and to compare services receiving core versus enhanced implementation strategies.

There was high perceived acceptability and appropriateness of ADAPT, with some concerns pertaining to impact on workload and time. Staff universally expressed support for addressing mental health in their service, and that ADAPT was evidence-based, could improve the quality of patient care, enhance staff experience of delivering care, and ultimately improve their service. However, staff at some sites found ADAPT to be too burdensome, unsuited to their patient population or unnecessary, suggesting lower levels of appropriateness. This was observed particularly in settings with a belief that adequate psychosocial care was already in place.

Our findings are in line with the Consolidated Framework for Implementation Research (CFIR) [23], which notes a number of characteristic of interventions that can impact their acceptability. These include the credibility of their source and evidence-base, relative advantage, complexity and cost, all raised by our participants. CFIR also emphasises the importance of organisational factors, including shared priority given to the intervention, and the centrality of patient needs. In our study, a culture of patient-centred care which valued attention to mental health was endemic, and articulated by participants as influencing the high perceived acceptability of ADAPT. The strong evidence-base and credibility of ADAPT’s University-based creators also resulted in high acceptability. ADAPT’s ability to ensure all patients with need were detected, and prevent morbidity escalation were strong factors contributing to perceived relative advantage.

This study also revealed staff and system factors that contributed to perceived relative advantage, factors perhaps less well articulated in the CFIR framework. Staff in this study felt that ADAPT delivered both the ability to deliver better care and to work within a multidisciplinary framework. Furthermore, reducing stress in patients also reduced their own work-related stress. At the health service level, ADAPT was seen as an opportunity to accrue data to support requests for additional psychosocial staff, to enhance interdisciplinary co-operation and expand referral networks. This suggests that implementation of an appropriate intervention (ADAPT CP) encourages further innovation and adaptation within services. These novel findings reinforce the importance of considering, planning for and measuring staff and service-level variables in intervention implementation studies. Within the CFIR framework, these are described as inner and outer setting variables.

A recent systematic review of hospital-based intervention implementation studies [24] noted that interventions which do not fit with existing workflows and processes, particularly if they require learning new information technology systems, can be more difficult to implement. Some of the challenges raised by our participants related to the process of implementation, that is, the sequence of steps required by the CP, which may have slowed down referral of patients with obvious need. However, CPs such as ADAPT are likely to be most beneficial in identifying patients who are *not* obviously in need, and are not intended to prevent usual care if greater speed is required. Perhaps a better articulation of how usual care and a new CP are intergrated, is required.

Despite enhanced sites being provided with additional support to recognise and address challenges to CP implementation as they arose, this did not impact perceived acceptability or appropriateness, with positive and negative views maintained over time, and with no discernable differences between study arms. This was apparent in both quantitative and qualitative data. This is surprising as the recent systematic review described above [24] noted that flexible interventions with ongoing tailoring and review (as was the case in enhanced ADAPT sites), can potentially overcome such difficulties. Thus these issues appear more contextually based. A detailed understanding of the local culture, resources and constraints at each service is required, some of which (such as staff turnover and changes) may not be readily changeable. Indeed, even with a modifiable periphery of elements within a CP, tailored to local preferences and context, some CPs may not have good fit for specific contexts, with alternative approaches required. It is important to learn from implementation efforts and co-design iterative versions of interventions with individual sites to ensure sustainability. Timing may be critical when introducing a new intervention also; some of our sites were undergoing organisational change and building works which made it more challenging to undertake a new CP.

This paper had a number of limitations. Thirteen services refused participation when approached, and thus potential bias through the inclusion of services with more interest in the ADAPT CP may have been introduced. However, as reasons for refusal were largely due to external influences (concurrent physical or organizational change) and there was considerable variability in participating services’ approaches to the ADAPT CP, we believe bias was minimal. While we obtained good multidisciplinary representation in a snapshot view of staff perceptions at each timepoint, staff movement meant that longitudinal data with a stable cohort was not possible. While interviewers were independent of the ADAPT research team who interacted with staff during implementation, social desirability bias may nonetheless have impacted responses, leading to a more positive perspective being presented than was actually felt. Due to more intensive interaction of ADAPT Team with Enhanced arm sites during implementation (e.g., during monthly meetings), there may have been issues impacting acceptability and appropriateness at Core services during the implementation phase to which we were not privy. Nevertheless, the qualitative analysis provided a rich and nuanced description of factors at the intervention, staff and service level impacting these outcomes.

## Conclusions

In conclusion, we found that ADAPT delivered an acceptable and appropriate program that enabled implementation of an anxiety and depression clinical pathway across 12 cancer services. Our study defined and operationalised these two implementation outcomes a priori and we were able to measure these over time using a robust combination of rich qualitative and quantitative data. Our findings reinforced that characteristics of the staff, service and its setting impact the acceptability and appropriateness of hospital-based interventions. Particular challenges are likely to be encountered in settings with high staff turnover and where continuity of care is more difficult to achieve, suggesting that adaptations of clinical pathways such as ADAPT will likely be required in such settings. Clinical pathways alone will not improve patient outcomes without effective and acceptable integration into existing workflows and service/system-level endorsement.

## Supplementary Information

**Additional file 1.** “CONSORT Flow Diagram”.

**Additional file 2.** “Management recommendations for each ADAPT CP step allocation”. A figure that describes which ADAPT CP step yields which intervention.

**Additional file 3.** “Acceptability/Appropriateness items”. A table outlining the thirteen study-developed items to assess acceptability/appropriateness.

**Additional file 4.** “Additional quotes related to acceptability/appropriateness”. A table providing additional quotes related to acceptability/appropriateness.

## Data Availability

Data and materials are available from the Corresponding author on request.

## Abbreviations

CP: Clinical Pathways
ADAPT CP: Clinical Pathway for the Screening, Assessment and Management of Anxiety and Depression in Adult Cancer Patients: Australian Guidelines
ADAPT: The Anxiety and Depression Pathway Program. A Translational Program Grant: A Sustainable and supported clinical pathway for managing anxiety and depression in cancer patients
ADAPT Portal: Web-based system to operationalise the Clinical Pathway for the Screening, Assessment and Management of Anxiety and Depression in Adult Cancer Patients: Australian Guidelines
CRCT: Cluster Randomised Controlled Trial
