# Supplementary figures and images for: Acceptability and appropriateness of a clinical pathway for managing anxiety and depression in cancer patients: a mixed methods study of staff perspectives
Source: BMC Health Serv Res. 2021 Nov 17;21:1243. doi: 10.1186/s12913-021-07252-z (PMC8600707; doi:10.1186/s12913-021-07252-z)

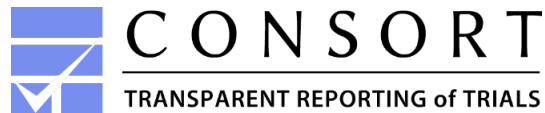

## CONSORT 2010 Flow Diagram

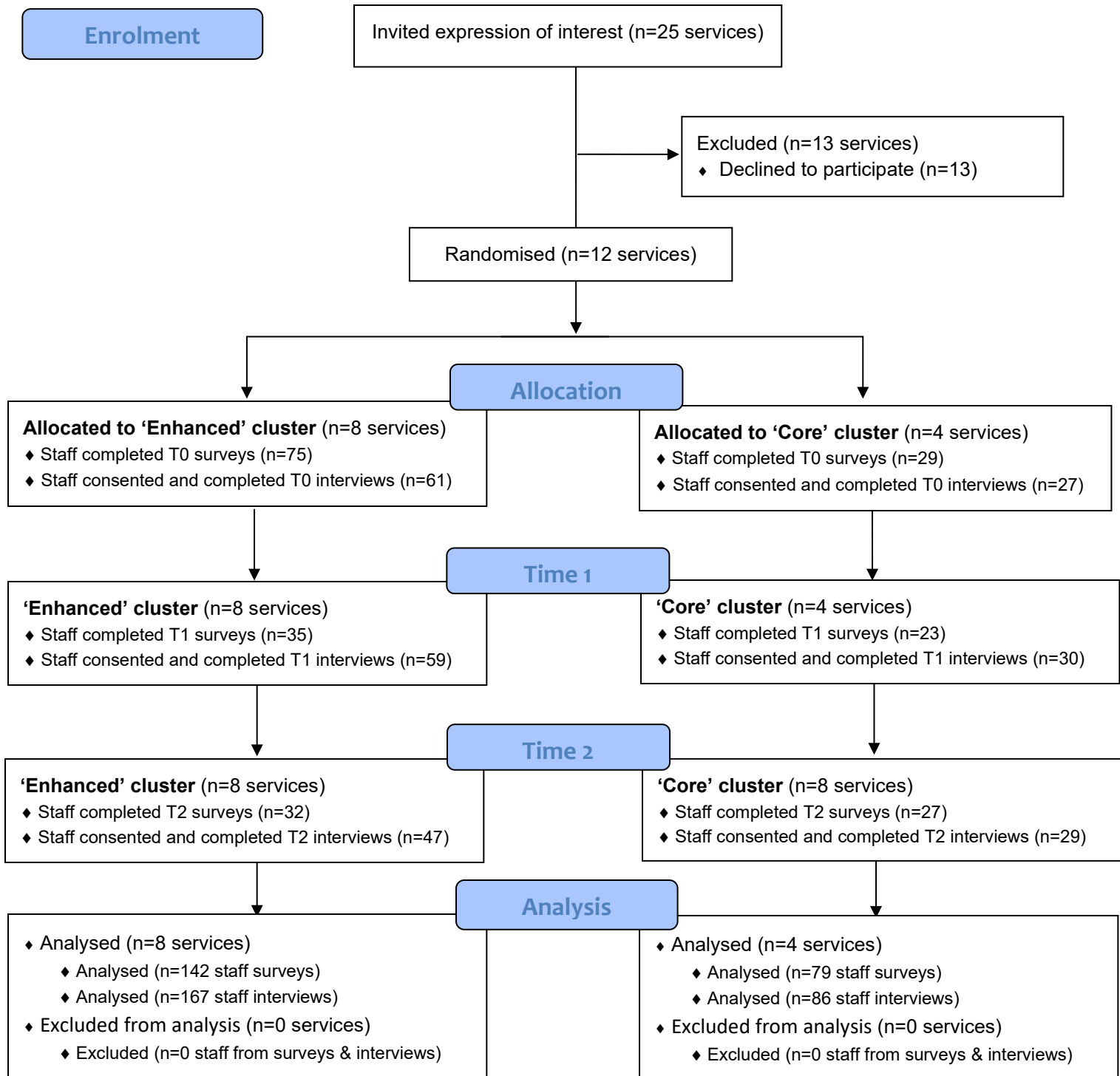

Supplement: Supplementary file 1 — Additional file 1. “CONSORT Flow Diagram”. [file 12913_2021_7252_MOESM1_ESM.pdf]

Additional File 2. Management recommendations for each ADAPT CP step allocation

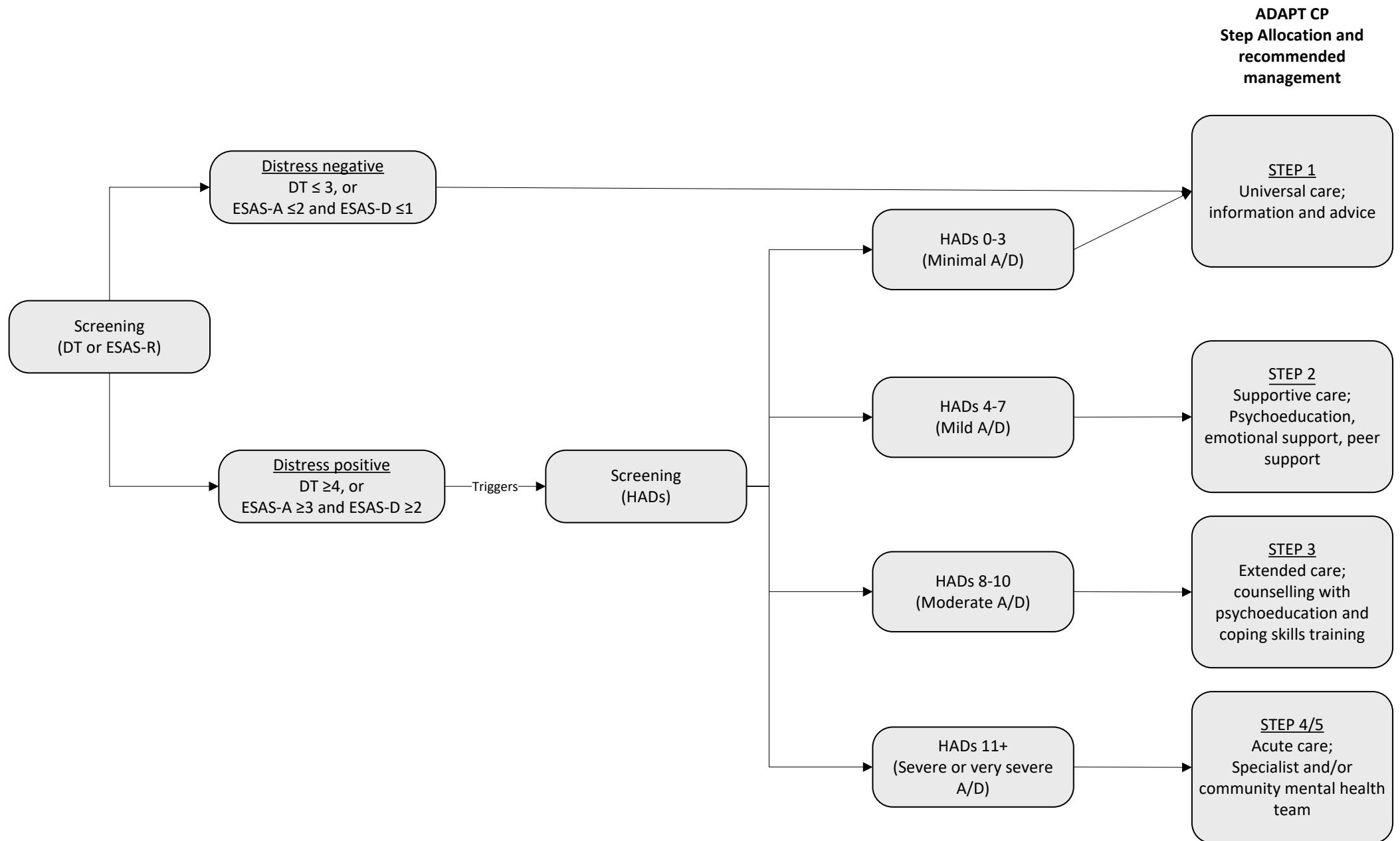

Supplement: Supplementary file 2 — Additional file 2. “Management recommendations for each ADAPT CP step allocation”. A figure that describes which ADAPT CP step yields which intervention. [file 12913_2021_7252_MOESM2_ESM.pdf]
