# Supplementary material for: Acceptability and appropriateness of a clinical pathway for managing anxiety and depression in cancer patients: a mixed methods study of staff perspectives
Source: BMC Health Serv Res. 2021 Nov 17;21:1243. doi: 10.1186/s12913-021-07252-z (PMC8600707; doi:10.1186/s12913-021-07252-z)
Supplement: Supplementary file 3 — Additional file 3. “Acceptability/Appropriateness items”. A table outlining the thirteen study-developed items to assess acceptability/appropriateness. [file 12913_2021_7252_MOESM3_ESM.docx]

Additional File 3. Acceptability/Appropriateness items#

| **Factor 1 – “Perceived benefit”** | **Factor 2 – “Perceived burden”** |
| --- | --- |
| 1. Patients in our local service would benefit from treatment for anxiety and/or depression | 7.^**^ Implementing the anxiety and depression pathway will increase my workload |
| 2. There is high quality evidence that psychological interventions can reduce anxiety in cancer patients | 8.^**^ Implementing the anxiety and depression pathway will take up too much of my time |
| 3. There is high quality evidence that psychological interventions can reduce depression in cancer patients | 12. I am confident we have the necessary staff to implement the anxiety and depression pathway |
| 4. The leaders in my organisation believe implementation of the anxiety and depression pathway is important | 13.^**^ I am confident we have enough resources to implement the anxiety and depression pathway |
| 5. The leaders of this organisation who are driving implementation of the anxiety and depression pathway have high credibility with me and I trust them |  |
| 9. I understand why the organisation needs to implement the clinical pathway for anxiety and depression |  |
| 11. The clinical pathway for anxiety and depression aligns with our organisation's mission and goals |  |
| 6*. The team evaluating the implementation of the anxiety and depression pathway have high credibility with me and I trust them | |

# One item (item 10: Implementing the anxiety and depression pathway will cost the organisation too much money) did not load on any factor and has been omitted.

* Item 6 loaded equally on both factors

** Items were reverse-coded.
