# Supplementary material for: Acceptability and appropriateness of a clinical pathway for managing anxiety and depression in cancer patients: a mixed methods study of staff perspectives
Source: BMC Health Serv Res. 2021 Nov 17;21:1243. doi: 10.1186/s12913-021-07252-z (PMC8600707; doi:10.1186/s12913-021-07252-z)
Supplement: Supplementary file 4 — Additional file 4. “Additional quotes related to acceptability/appropriateness”. A table providing additional quotes related to acceptability/appropriateness. [file 12913_2021_7252_MOESM4_ESM.docx]

Additional File 4. Additional quotes related to acceptability/appropriateness

| **Theme** | **Subtheme** | **Quote** |
| --- | --- | --- |
| **Mental health important, which ADAPT addresses** |  | *“I grabbed a few research articles that indicated that the gold standard of treatment for cancer patients was, um, to screen for anxiety and depression because it’s pretty prevalent in that population”* (E_PSYCH_S06P04T0)  *“I think with our staff, everyone's definitely on-board about tackling those kinds of issues and addressing them*.” (E_NURS_S07P06T1) |
|  | **Our responsibility** | *“I definitely think it’s needed. I, I think it’s something that’s been probably missing for a really long time”* (C_NURS_S12P01T0)  *“Yeah definitely, I think, I mean any psychological support that patients can get is very valuable, yes.”* (C_NURS_S03P05T2) |
|  | **Evidence-based** | *"And I thought ADAPT - it’s a comprehensive research program*.”(E_PSYCH_S04P06T0) |
|  | **Systematic and ensure patients not missed** | *“[If all patients are screened] it’s more of a level playing field, you know. Somebody is not going to miss out on an evaluation or then an important referral because someone is absent.”* (E_NURS_S05P08T2) |
|  | **Nip problems in the bud** | “*I think people are expecting to see, more people be seen by the psycho-oncology counsellor, and probably also has a lot of issues picked up at an earlier stage, that people don’t present with suicidality or, um, very dire consequences with their depression, as a result of cancer.*” (E_MED_S05P09T1) |
|  | **Empower patients to self-manage** | *“And I think there is still stigma around mental health, which means that a lot of patients were saying, “I’ll deal with it myself; I’ll speak to my GP; I’ll speak to my family,” but they’re not really doing any of those things. So I think, having ADAPT as a routine and normal part of clinical practice reassures patients that it’s okay to express that they are anxious or depressed and to therefore be linked in with the services earlier on.”* (E_MED_S06P12T0) |
| **ADAPT supports staff** |  | *“I think it’s been – I think it’s a really good idea that we have this program to make us stop and think about that part of holistic care.”* (E_NURS_S05P05T0)  “*One thing I like about ADAPT is that… it’s a much more structured process of… once we get the information from the patient, how to deal with it, and it’s a lot more clear cut for the staff to carry out*.” (C_ADMIN_S03P01T0)  *“Getting them to the appropriate care, when to intervene, who to intervene with or what intervention to intervene with. So, having a pathway that has all those factors that are pre-determined, makes it easier for individuals to make sure that the patient gets the right care*.” (C_MED_S01P09T2)  *“So to have something… they can flag to us that there’s an issue… and then we can… say, oh, look, you’ve put this down on your survey, what’s that about*?”*… to see, what are the actual main concerns you know, are they physical, or they're work, or even coming down to like sexuality, and relationships. That’s not necessarily something we would speak of, unless prompted by the patient. But, once they have that positive screening, you can then kind of have a discussion around those issues.”* (E_NURS_S04P09T1)  *“So it's fantastic that it has increased awareness for the nursing staff and the doctors of being able to identify patients in need of psychosocial support… So it's good that ADAPT is there now*.” (C_PSYCH_S12P02T2) |
|  | **Multidisciplinary approach supportive and enriching** | *“So, what I feel is that ADAPT will give us a way of working smarter, in that it should help identify the people who really do need to see a clinical psychologist… so I really like the triage aspect.”* (E_PSYCH_S06P04T0)  *“I guess also working collaboratively with the other members of the MDT on something like this was quite positive*.” (E_PSYCH_S10P04T2)  *“So this has actually given them [administrative staff] something really nice… they feel like they have been a part of something… that is helping us all out*.” (C_NURS_S01P04T0)  *“And on a personal level, it’s enabled me to get to know better, a lot of people who work in the area generally, just because ADAPT has been a project that pulls on resources from different areas within the department.”* (E_PSYCH_S06P04T2) |
| **ADAPT is fit for purpose** | **Appropriateness for staff** | *“Uh, I think, um, I think initially it will be, um, uh, a little bit of extra work but… I think in the long run, it will hopefully decrease some of the hours that we spend sitting with people discussing these things.*” (C_ NURS_S01P04T0)  “*I think people think it’s all a good idea in theory, but when you actually have to do the work. You know, and incorporate it in your usual work demands. It all becomes too hard for a lot of people.*” (E_PSYCH_S10P01T2)  “*at the end, they were running out of interest in it. They were running out of puff*… *We were saying “yeah, but it’s got value,” but thank goodness, we’ve got so much to do… it’s just another thing, you know*” (C_ADMIN_S01P07T2)  *“Everyone that I have spoken to sort of feels like it’s a good in theory idea. Um, but the main concern is about increasing people’s workload, or that, yeah, we don’t have the staff to--- actually roll it out*” (E_PSYCH_S06P08T2)  “*It didn't seem like we had that many referrals and there seemed to be a lot of barriers and not much of a way forward with it*.” (E_ADMIN_S04P01T2) |
|  | **Appropriateness for patients** | *“I’ve found with a lot of patients they don’t want to bring it back up I suppose. It’s something that’s in the past, so they don’t want to deal with it… A lot of them are still very rigid on not wanting to progress with the pathway or assessment”* (C_NURS_S03P07T2)  “*The fact that we couldn’t register any non-English speaking patients. At least 30% of our patient load is non-English speaking people so there's a big chunk of people that don't even get that option to be a part of it*” (C_NURS_S01P04T2)  “*For the bulk of the ones that need the help, it's just their complete inability to use computers. But they just kind of freak out a little bit and they just, kind of, go deer in the headlights*”. (C_NURS_S01P06T2) |
| **ADAPT: a catalyst for change** | **System improvement, additional resources** | “*If the outcome does mean that we do get more psychology service, or psychologist service for the patients, then that would be ideal.*” (E_NURS_S04P03T0)  “*It would be able to tell us at different points where people are more likely to have higher levels of anxiety and depression… to be able to improve– the way we work, and also improve our outcomes for patients*.” (E_PSYCH_S10P04T0)  *“I remember thinking at the time this would be an excellent way to get some sort of [psycho-oncology] service up and running and so subsequently we had a booking for the psycho-oncology position.”* (E_MED_S05P09T0)  “*But the truth is if you have… very robust data showing that we’re screening a large cohort of patients and 30% of them have significant distress or psychosocial issues that would warrant referral to a psycho-oncologist, and you could crunch out the numbers and say, well, that would justify hiring another three extra psychologists or social workers, so it can actually very much work to your advantage using the process to lobby for additional resources*” (C_MED_S11P05T1) |
